# Knowledge, Attitudes, and Usage of Apitherapy for Disease Prevention and Treatment among Undergraduate Pharmacy Students in Lithuania

**DOI:** 10.1155/2015/172502

**Published:** 2015-11-30

**Authors:** Sonata Trumbeckaite, Jurgita Dauksiene, Jurga Bernatoniene, Valdimaras Janulis

**Affiliations:** ^1^Department of Pharmacognosy, Faculty of Pharmacy, Academy of Medicine, Lithuanian University of Health Sciences, Eiveniu Street 4, LT-50009 Kaunas, Lithuania; ^2^Department of Drug Technology and Social Pharmacy, Faculty of Pharmacy, Academy of Medicine, Lithuanian University of Health Sciences, Eiveniu Street 4, LT-50009 Kaunas, Lithuania

## Abstract

Traditional medicine therapies are historically used worldwide for disease prevention and treatment purposes. Apitherapy is part of the traditional medicine based on bee product use. Complementary medicine practices which incorporate use of some traditional herbal, mineral, or animal kind substances very often are discussed with pharmacy professionals because these products are often sold in pharmacies as dietary supplements. This study is aimed at determining the attitude, knowledge, and practices of apitherapy among undergraduated pharmacy students (Master of Pharmacy) who already have a pharmacy technician diploma and from 1 to 20 years of practice working in a community pharmacy as pharmacy assistants. A method of questionnaire was chosen. The questions about attitudes, experience, knowledge, and practices for disease prevention and treatment of different bee products, their safety, and informational sources were included. Respondents shared opinion that use of bee product is part of the traditional medicine. Most of them had experience on honey product use for treatment and disease prevention for themselves and their family members (62%) although the need of more evidence based information was expressed. The most known bee products were honey, propolis, and royal jelly. They are widely used for enhancing the immune system and prevention of respiratory tract infection.

## 1. Introduction

Apitherapy (*Apis* is a Latin word that means bee) is the practice of using bee products such as honey, pollen, propolis, royal jelly, and bee venom for disease prevention or treatment proposes. It can be also described as “the science (and art) of the use of honeybee products, to maintain health and assist the individual in regaining health when sickness or accident interferes” [1, 2].

In the past, the apitherapy products were frequently used as natural remedies for health maintenance. In many countries, bee products are part of traditional medicine. The roots of apitherapy can be traced back to more than 6000 years of medicine in ancient Egypt. The ancient Greeks and Romans also used bee products for medicinal purposes. There is also evidence that honey was part of traditional Chinese medicine: The famous ancient prescription book with fifty-two prescriptions dating back to the third century B.C. found in Changsha, Hunan Province, contains two prescriptions involving bees, one of which uses honey to treat diseases [1–4].

More recently, the bee products have been incorporated into modern medical practice, where the focus of attention is mainly the illness and its prevention [5]. Among the complementary and alternative medicine (CAM) modalities, some dietary supplements show relatively strong positive evidence for being effective in the prevention of some common diseases [6, 7]. Some findings suggest that those who use alternative therapies, including herbal, mineral, and biological (including apitherapy) dietary supplements, appear less likely overall than nonusers to receive standard preventive care. In addition, users of dietary supplements are more likely to engage in healthy behaviours and appear to be a more health-conscious group [8].

Lithuania has very old beekeeping traditions and for centuries bee products have been used in folk medicine for treatment of wounds, cough, ulcers, tuberculosis, and other diseases. Scientific research on apitherapy in Lithuania started more than 50 years ago when fresh royal jelly was applied at the Cardiology Department for patients suffering from cardiovascular diseases [2, 9]. From 1971, Lithuanian scientists have been focusing on investigation of propolis qualities and propolis preparations development. Nowadays, bee products, particularly honey and propolis and its preparations (tablets, suppositories, ointments, mouth sprays, and others), are quite popular among consumers and are available in most of the Lithuanian community pharmacies usually positioned as dietary supplements.

The changing role of pharmacists encourages them to discuss with pharmacy patients not only the correct usage of medication but also disease prevention and public health issues. The provision of information on disease prevention is part of the public health issues and may empower people to increase control over and to improve their health [10, 11].

The community pharmacists are recognized as the most accessible to the public health care professionals. They are like gatekeepers when giving advice for patients who enter community pharmacy to obtain medication, dietary supplements, or medicine goods [11]. In Lithuania, like in many other countries, the community pharmacy is the only legal place to obtain medication and the pharmacist consultation is an obligatory part of the purchase [12]. The patients who make decisions about their health often use integrative practice and combine both conventional and complementary medicine practices [6, 8]. Complementary medicine practices which incorporate use of some traditional herbal, mineral, or animal kind substances very often are discussed with pharmacy professionals because these products are often sold in pharmacies as dietary supplements. Pharmacy professionals often encourage people to use dietary supplements because (1) there is no prescription need for them, (2) this gives additional income for pharmacy, and (3) pharmacists value their knowledge on dietary supplements. Dietary supplements are often used for disease prevention purposes [13, 14]. The correct knowledge on common complementary medicines practices is necessary to assist patient needs and answer the questions. This includes providing information that allows the patient to make the informed decisions about their health.

The aim of this study was to investigate the experience, knowledge, attitude, and practices of undergraduate pharmacy students towards bee products for disease prevention and treatment purposes.

## 2. Methods

### 2.1. Setting and Sample

This study was conducted among undergraduated pharmacy students at the Lithuanian University of Health Sciences (LUHS). The sample of undergraduate Master of Pharmacy (MPharm) intensive course students was chosen for the cross-sectional study. All participants of intensive MPharm course already have a Professional Bachelor degree as pharmacy technicians and most of them declared from 1 to 20 years of practice in community pharmacy as pharmacist's assistants (the demographic characteristics of the sample are presented in Table 1). In Lithuania, pharmacy technicians after registration in the List of Pharmacy Assistants at Lithuanian State Medicines Control Agency can work in community pharmacies as pharmacist's assistants and consult pharmacy patients under pharmacist's supervision [12].

From the year 2013, the curricula of MPharm program of Lithuanian University of Health Sciences have a special course on integrated evidence based CAM education. The course is given to the last year students before graduation.

### 2.2. Data Collection

Data were collected by cross-sectional survey. The questionnaires were distributed during the 2014-2015 academic year at the Lithuanian University of Health Sciences to the intensive 3-year MPharm course students. The respondents of all three years were invited to take part in a survey. Third-year students answered questions before the beginning of special course on integrated evidence based CAM education on use of dietary supplements with a special focus on traditional Lithuanian medicine: herbal, mineral, and biological (including bee products) dietary supplements. This ensured that the answers were not influenced by lectures of apitherapy and expressed earlier students' attitude, knowledge, and practice of bee product use.

The questionnaires were distributed to each student together with a written consent form with the explained aim of the survey and they were informed of confidentiality. All participants were assured that their refusal to participate in the research will not have any influence on their evaluation grades during upcoming courses. The signature on the consent form was accepted as an indicator that a student wishes to participate in a research. It took from 10 to 20 minutes for every participant to fill up the questionnaire. All filled forms were collected by the class leader and returned to the leading investigator. The return rate of the questionnaire was more than 90%.

### 2.3. Study Questionnaire

The questionnaire consisted of 8 sections. Section 1 consisted of questions about general attitudes toward apitherapy, everyday personal practices, and informational sources. Sections 2–6 were formed up with questions about knowledge and practices for disease prevention and treatment for different bee products: honey, propolis, royal jelly, bee pollen/bee bread, and bee venom. Section 7 consisted of attitude towards apitherapy safety questions. The last section was with questions for demographic characteristics of the respondents. Most of the questions were closed, with the proposed choices of answers with “tick” box possibilities. Likert scale was used to evaluate the general attitude towards apitherapy and disease prevention practices among respondents with different bee products.

### 2.4. Data Analysis

SPSS (version 22.0) was used for data analysis. Descriptive statistics such as frequencies, means, and ranges were calculated to summarize the data. For the Likert scale responses, all responses with any level of degree of agreement were grouped together as positive responses and all responses with any degree of disagreement were grouped as negative respondent. For knowledge and use of bee products for disease prevention, the Likert scale responses were transformed into Index score which was calculated as an average of all responses of 5-point Likert scale evaluation (5: strongly agree; 4: agree; 3: neutral; 2: disagree; 1: strongly disagree). *t*-test and chi-square were used to analyze the differences among groups. Results were considered significant when the *p* value was less than 0.05.

## 3. Results

### 3.1. Pattern of Awareness and Use of Apitherapy

All 72 (100%) respondents indicated that they use and are aware of at least one of the bee products. Honey was the most popular choice for all indicators (“I know”; “I use by myself and encourage my family members to use for treatment purposes”; “I use by myself and encourage my family members to use for disease prevention”; “I recommend it to pharmacy patients for treatment”; “I recommend it to pharmacy patients for disease prevention”). More than half (62%) were convinced of the usage of bee products by themself and almost one-third (34%) reported recommendation to pharmacy patients. The most popular choice for “disease prevention” purposes was “honey” for family members (28%) and propolis (16%) or “royal jelly” (13%) for pharmacy patients. “Bee venom” was the rarest choice for all statements. It was mentioned only by 4 respondents.

### 3.2. Attitudes towards Apitherapy

In Table 2, the attitudes of the respondents towards apitherapy are summarized. Most of the respondents think that apitherapy is part of the Lithuanian traditional medicine and as future pharmacists they must have sufficient knowledge on apitherapy, but only 10% confirmed themselves already as apitherapy experts. The statistical analysis demonstrated that more confident about their apitherapy knowledge are those who are older than 26 years, who have more than 5 years of experience working as pharmacist assistant and also those who have beekeepers among their parents or expect their children to be beekeepers (*p* < 0.05). There was no significant difference among other demographic groups although students of third year and those who declared to have beekeepers among their grandparents rated their knowledge as sufficient. All 7 respondents who declared themselves as experts of apitherapy had beekeepers among their ancestors and more than 5 years of working practice as pharmacy assistant. More than half (72.2%) agreed that apitherapy products should be available at every community pharmacy whereas only 5.6% disagree. About the extra question whether the pharmacist should promote apitherapy, 69% of all respondents answered positively, and the rate was higher in beekeepers among ancestors group (89%).

### 3.3. Sources of Information on Apitherapy

In Table 3, the most important sources of information on apitherapy are presented. The main sources were the Internet (62.2%), journals (59.7%), and formal lectures of continuing education (52.8%). Even 41.7% said that parents and grandparents were source of information on apitherapy. The respondents expressed opinion that health care providers as pharmacists (61.1%) and physicians (47.2%) should provide information on apitherapy and only 26.4% said that it should be done by traditional healers.

### 3.4. The Knowledge and Use of Bee Products for Disease Prevention

The undergraduate pharmacy students experience on knowledge and use of bee products for disease prevention is summarized in Table 4. Thus, only “enhancing immune system” and “respiratory tract infections” (all four types of bee products: honey, propolis, royal jelly, and bee pollen/bee bread, except for bee venom) were evaluated with Index average more than 3—this means that most of the participants believe honey products to be effective for this purpose. Respondents thought that, for prevention of respiratory tract infection, the best among bee products are honey (the range of the scale 3.57, i.e., 62.5% “strongly agree”) and propolis (the range of the scale 3.42, i.e., 53.5% “strongly agree” and about 30% of respondents “agree”).

Bee pollen also got high cumulative Index score of 3 for increasing male and female fertility. Also, the respondents demonstrated positive attitude towards the bee products (except for bee venom) usage for fertility increasing proposes (40.6%, 34.3%, 38%, and 34% agreed that honey, bee pollen, royal jelly, and propolis might help, resp.). Bee venom has not been considered by respondents as a possible product for prevention of all indicated areas in the table.

Respondents did not have much experience and knowledge on bee product use for such prevention areas as cardiovascular diseases, cancer, and disorders of endocrine system. The range of scale for the usefulness of various bee products (honey, propolis, royal jelly, and bee pollen/bee bread) varied between 1.8 and 2.54. Thus, about 60% of respondents did not agree that these products could be used for prevention of cardiovascular diseases, cancer, or endocrine system disorders.

Regarding prevention of skin aging, only about 20% of the respondents strongly agree and about 30% agree that bee products, honey, propolis, and royal jelly, could be helpful whereas more than 40% disagree.

### 3.5. The Knowledge and Use of Bee Products for Treatment of Diseases

Table 5 shows that bee products such as honey, propolis, royal jelly, and bee bread are widely used and recommended to pharmacy patients. The main indication is respiratory tract infections: Every third respondent would use and recommend to the pharmacy patients honey or propolis as main therapy and more than half of all respondents choose honey, propolis, royal jelly, and bee bread as an additional therapy. Only less than 10% of the respondents indicated “no use” or “no knowledge” about honey use for respiratory tract infections. 37.1% of the respondents indicated bee venom as a main therapy among all bee products for treatment of arthritis and 22.9% as an additional therapy.

According to 22.1% of undergraduate pharmacy students for skin diseases propolis could be a main therapy among bee products and according to 55.9% an additional therapy. 53.7% of the respondents believed honey and 44.9% propolis as an additional therapy for herpes treatment and only 16% bee venom. About 34–36% thought that royal jelly and bee pollen could be used as an additional therapy.

### 3.6. Safety Issues of Apitherapy Products

The experience and knowledge on safety issues of bee products are presented in Table 6. Participants of the study indicated that bee products have less contraindications than other remedies. 48.7% of the respondents agree or strongly agreed with this statement and none (*N* = 0) strongly disagreed (Table 2). They also indicated the attitude about fewer side effects than conventional remedies. The most known side effect is allergy (97.2%) and bee product use should be recommended with warnings to allergic patients (90.3%), pregnant woman (61.1%), or children under 3 years of age (62.5%).

## 4. Discussion

The undergraduate pharmacy students of LUHS who have already from 1 to 20 years of experience as consulting pharmacy assistants demonstrated a positive attitude towards apitherapy. According to them, apitherapy is part of the tradition medicine though not so very popular nowadays. Still they are positive about having a wide spectrum of bee products at community pharmacies. 62% of the respondents reported the use of bee products for themselves or their family members. This repeats the results of pharmacy students surveys towards attitude and use of complementary and alternative medication in Australia [15], Great Britain [16], Kuwait [17], Malaysia [18], and Sierra Leone [19]. In an Australian survey [15], about 90% of all-years students declared that clinical care should integrate the best of conventional and CAM practices; 60% of undergraduate British pharmacy students stated that they were very interested in complementary and alternative medicine [16]; 79.7% of Kuwait students believed that CAM includes ideas and methods from which conventional medicine could benefit [17]; 77.6 of the Malaysian study participants had used CAM previously [18]; and 55.6% of Sierra Leones respondents indicated that CAM therapies are effective and not harmful [19]. The comparable studies in USA [20] or Germany [21] made with undergraduate medical students also demonstrated positive attitudes towards CAM: medical students in USA survey declared earlier experience of a wide spectrum of CAM modalities [20] and almost 68% of German medical students indicated “earlier experience” as “source of information” for knowledge on CAM [21]. WHO supports the idea about integration of conventional and complementary practice in order to reach the best results for the patient and society [5].

The respondents of our study more often tend to use the apitherapy by themselves rather than offering it to the pharmacy patients. The same findings were in USA study [20] where more students used herbs or supplements rather than recommending herbs or dietary supplements to the patients. Some studies conclude that, due to the current popularity of complementary and alternative medicine (CAM) among patients, many pharmacists will be faced with questions from the public regarding natural products and other CAM therapies and there is a great need for exact knowledge [13, 14].

In our study, the most important sources of information on apitherapy were the Internet (62.5%), journals (59.7%), and formal lectures of continuing education (52.8%). Even 41.7% obtained information from parents and grandparents and only 18% from media. In a German [21] study, the “practical experience” (68%), media (48%), and also “other publications and congresses” and “medical education” were named. Pharmacy students of Sierra Leone listed media (58.9%), books (35.6%), and CAM practitioners (43.3%) [19]. Moreover, the results of the survey revealed that undergraduate pharmacy students believe that one of the main information sources to the public about apitherapy should be pharmacist (62.5%) or physician (47.2%). 80.6% of the respondents have chosen “apitherapist” as the answer for this question but there is no apitherapists activity regulation in Lithuania. The choice of health care providers as expected source for information on apitherapy also reports the need of evidence based information integrated in the pharmacy program curriculum. It was observed also in other studies [22].

Most of our respondents think that pharmacist should have sufficient knowledge towards apitherapy but only a small part of them think about themselves as experts. This strongly correlates with traditions of beekeeping in the family [2, 9]. The German survey of beekeepers [3] showed that most of them had positive experience in using honey, propolis, pollen, and royal jelly which they employ for various indications. Common cold, wounds, sore throat, and gingivitis were one of the most often mentioned indications for treatment purposes. In our survey, more than 90% of respondents indicated the use of honey for respiratory tract infections as main or additional therapy. Also, propolis and bee pollen were indicated. All bee products were also chosen for prevention reasons. “Enhancing immune activity” and “prevention of respiratory tract infection” were also very popular choice of our respondents as area of all bee products use except bee venom.

Propolis has been reported to exert a wide range of biological activities: antibacterial, antiviral, anti-inflammatory, an immunomodulatory properties as demonstrated in* in vitro* and* in vivo* studies [23, 24]. Nowadays, propolis and its preparations in various forms for use (mouth sprays, tablets, capsules, etc.) are used in human medicine to treat common cold, flu-like infections, wounds, sore throat, and herpes simplex infection [24, 25].

According to the study of Paul [26], “honey may be a preferable treatment for the cough and sleep difficulty associated with childhood upper respiratory tract infection.” Honey is used as a common ingredient or alone in folk medicine for relieving of cough. Royal jelly and bee pollen/bee bread were also mentioned by respondents among other bee products to enhance the immune system. It has been shown that royal jelly possesses immunomodulatory activity [27, 28], antioxidant properties [29], and antimicrobial activities [30].

Regarding bee products for prevention of cardiovascular or cancer diseases, the students did not express united position: 29–40% of the respondents agreed or strongly agreed that propolis, honey, royal jelly, or bee pollen could be useful for disease prevention purposes and, respectively, 24–32% for cancer prevention. Current* in vitro* studies show a potential of selective bee products against tumor cells [31, 32].

Regarding bee venom, 60% of undergraduated pharmacy students think that in case of arthritis bee venom could be used as a main or additional therapy; however, they reported no or less knowledge on bee venom therapy for other listed diseases. Bee venom is mostly known as anti-inflammatory and pain-reducing agent and, in form of bee stings, apipuncture, injections, and so forth, is used by apitherapy practitioners in some countries to treat arthritis [4] or other diseases, but there is still a great need for evidence based knowledge.

An important factor by using bee products is human safety. Most of our respondents named allergy as the main possible side effect and also stated that generally allergic patients should avoid this therapy. This fact is supported by other studies [33, 34].

## 5. Conclusions

Pharmacy students in Lithuania showed interest towards bee product use for diseases prevention and treatment purposes. They self-reported use and awareness of apitherapy products which are part of traditional medicine in Lithuania. According to them, the pharmacist as the easiest accessible health care professional is the one who can support pharmacy patients with appropriate information on apitherapy while apitherapy products are in most of the community pharmacies. Enhancing immune system and prevention and treatment of the respiratory tract infections were the main areas of bee product use.

## Figures and Tables

**Table 1:** Sociodemographic characteristics of the respondents (*n* = 72).

Demographics	*n* (%)
Year of study	
First year	23 (31.9)
Second year	23 (31.9)
Third year	26 (36.2)
Gender	
Male	10 (13.9)
Female	62 (86.1)
Age group	
≤22	14 (19.4)
22–26	38 (52.8)
27–32	3 (4.2)
≥33	17 (23.6)
Birth place	
City	47 (65.3)
Town	19 (26.4)
Countryside	6 (8.3)
Work place	
City	62 (13.9)
Town	10 (86.1)
Countryside	0 (0)
Work experience at the community pharmacy	
≤1 year	18 (25.0)
1–4 years	34 (47.2)
≥5 years	17 (23.6)
No experience	3 (4.2)
Experience and expectation towards beekeeping of the family members	
Presence of beekeepers among parents	
Yes	5 (6.9)
No	66 (91.7)
I am not sure	1 (1.4)
Presence of beekeepers among grandparents	
Yes	15 (20.8)
No	50 (69.5)
I am not sure	7 (9.7)
Expectation towards children beekeeping	
Yes	9 (12.5)
No	41 (56.9)
I am not sure	22 (30.6)

**Table 2:** The attitude towards apitherapy.

Statement	Strongly agree	Agree	Neutral	Disagree	Strongly disagree
	(%)	(%)	(%)	(%)	(%)
Apitherapy is part of our traditional medicine	31.4	42.9	24.3	1.4	0
Apitherapy is very popular nowadays in our country	0	16.7	43.1	37.5	2.8
Physicians have sufficient knowledge on apitherapy	0	8.5	36.6	52.1	2.8
As a future pharmacist I have sufficient knowledge on apitherapy	45.7	35.7	18.6	0	0
As a pharmacist I am an apitherapy expert	0	9.9	29.6	46.5	14.1
Apitherapy has less contraindication than other remedies	6.9	36.1	45.8	11.1	0
Apitherapy has less side effects than other remedies	5.6	43.1	43.1	8.3	0
The use of apitherapy products should be encouraged	11.1	36.1	47.2	5.6	0
Apitherapy products should be available in every community pharmacy	26.4	45.8	22.2	4.2	1.4

**Table 3:** Sources of information.

Question	(%)
What are the main sources of the information on apitherapy for you?	
Parents/grandparents	41.7
Friends/community members	9.7
Journals	59.7
Internet sources	62.2
Other health professionals	5.6
Media	18.1
Formal lectures	52.8
Other	11.1
Who is supposed to be the main informational source on apitherapy to the patients?	
Physician	47.2
Pharmacist	61.1
Beekeepers	44.4
Traditional healers	26.4
Apitherapist	80.6
Scientists	20.8
Other	1.4

**Table 4:** The knowledge and use of bee products for disease prevention.

Prevention area	Honey	Propolis	Royal jelly	Bee pollen and bee bread	Bee venom
Enhancing immune system	3.86^*∗*^	3.54	3.46	3.48	2.01
Respiratory tract infections	3.57	3.42	3.16	3.2	1.98
Cardiovascular diseases	2.02	2.2	2.4	2.28	1.66
Cancer prevention	1.9	2.1	2.03	2.03	1.71
Endocrine system disorders	1.82	1.93	2.1	2.14	1.68
Allergy	1.36	1.68	1.73	1.6	1.55
Skin aging	2.54	2.51	2.42	2.27	1.57
Anemia	1.98	2.11	2.2	2.18	1.55
Increasing of male/female fertility	2.25	2.12	2.24	3	1.55
Enhancing mental activity	2.64	2.48	2.48	2.34	1.61

^*∗*^Index score is calculated as an average of 5-point Likert scale evaluation (5: strongly agree; 4: agree; 3: neither agree nor disagree; 2: disagree; 1: strongly disagree). The max Index meaning is 5 and minimum is 1.

**Table 5:** The knowledge and use of bee products for treatment.

Treatment area	Honey	Propolis	Royal jelly	Bee pollen and bee bread	Bee venom
	(%)	(%)	(%)	(%)	(%)
Arthritis					
Main therapy	4.7	6.0	3.1	4.5	37.1
Additional therapy	39.1	37.3	32.3	26.9	22.9
No knowledge	43.7	47.7	58.5	59.7	32.9
No use	12.5	9.0	6.2	9.0	7.1
Respiratory tract diseases					
Main therapy	29.8	34.8	15.7	19.1	4.4
Additional therapy	62.7	52.2	53.1	54.4	23.5
No knowledge	4.5	11.6	28.1	23.6	60.3
No use	3.0	1.4	3.1	2.9	11.8
Skin diseases					
Main therapy	18.4	22.1	10.8	13.2	4.4
Additional therapy	55.4	55.9	46.1	33.9	29.0
No knowledge	23.1	20.5	40.0	50.0	53.6
No use	3.1	1.5	3.1	2.9	13.0
Gastrointestinal disorders					
Main therapy	10.1	13.4	12.3	9.8	1.5
Additional therapy	52.2	41.8	38.5	42.3	13.6
No knowledge	33.3	35.8	44.6	42.3	71.3
No use	4.4	9.0	4.6	5.6	13.6
Tuberculosis					
Main therapy	1.5	1.5	1.5	2.9	0.0
Additional therapy	28.8	28.8	23.5	19.1	11.9
No knowledge	53.0	59.1	66.2	67.6	73.2
No use	16.7	10.6	8.8	10.4	14.9
Oncology					
Main therapy	1.5	4.5	1.5	2.9	1.5
Additional therapy	36.8	25.4	24.6	20.6	14.7
No knowledge	47.0	61.1	65.2	64.7	70.6
No use	14.7	9.0	8.7	11.8	13.2
Anemia					
Main therapy	4.4	4.5	5.8	4.3	0.0
Additional therapy	36.7	25.8	29.0	31.9	12.0
No knowledge	51.5	60.6	62.3	56.5	74.6
No use	7.4	9.1	2.9	7.3	13.4
Herpes					
Main therapy	6.0	11.5	4.5	1.5	0.0
Additional therapy	53.7	44.9	34.3	36.2	16.2
No knowledge	32.8	37.7	55.2	56.5	70.6
No use	7.5	5.8	6.0	5.8	13.2
Gynecological inflammations					
Main therapy	1.5	3.0	0.0	4.3	1.5
Additional therapy	26.5	27.3	20.9	21.8	11.7
No knowledge	58.8	59.1	74.6	66.7	72.1
No use	13.2	10.6	4.5	7.2	14.7
Benign prostatic hyperplasia					
Main therapy	0.0	0.0	0.0	2.9	2.9
Additional therapy	19.4	21.5	17.9	17.4	10.3
No knowledge	67.2	63.1	76.1	72.5	70.6
No use	13.4	15.4	6.0	7.2	16.2
Ophthalmologic disorders					
Main therapy	6.0	15.1	3.1	0.0	0.0
Additional therapy	46.3	37.9	18.5	21.2	0.0
No knowledge	40.3	39.4	72.3	68.2	64.3
No use	7.5	7.6	6.1	10.6	35.7

**Table 6:** Safety issues of bee products.

Question	(%)
What group of patients should not use the bee products?	
Pregnant women	61.1
Children under 3 years of age	62.5
Teenagers	18.1
Oncology patients	15.3
Allergic patients	90.3
≥65 years of age	1.4
Other	4.2
What are the possible side effects of the bee products?	
Allergy	97.2
Bleeding	2.8
Headaches	13.9
Weight loss or increase	1.4
Vomiting	45.8
Vision disorders	0.0
Other	0.0
